# Natural and iatrogenic ocular manifestations of rheumatoid arthritis: a systematic review

**DOI:** 10.1007/s10792-021-02058-8

**Published:** 2021-11-21

**Authors:** Rosanna Dammacco, Silvana Guerriero, Giovanni Alessio, Franco Dammacco

**Affiliations:** 1 Department of Ophthalmology and Neuroscience, University of Bari “Aldo Moro”, Medical School, Bari, Italy; 2 Department of Biomedical Sciences and Human Oncology, University of Bari “Aldo Moro”, Medical School, Polyclinic, Piazza Giulio Cesare 11, 70124 Bari, Italy

**Keywords:** Rheumatoid arthritis, Ocular adverse drug reactions, Causality in adverse drug reactions, Disease-modifying antirheumatic drugs, Non-steroidal anti-inflammatory drugs, Tumor necrosis factor inhibitors

## Abstract

**Purpose:**

To provide an overview of the ocular features of rheumatoid arthritis (RA) and of the ophthalmic adverse drug reactions (ADRs) that may be associated with the administration of antirheumatic drugs.

**Methods:**

A systematic literature search was performed using the PubMed, MEDLINE, and EMBASE databases. In addition, a cohort of 489 RA patients who attended the Authors’ departments were examined.

**Results:**

Keratoconjunctivitis sicca, episcleritis, scleritis, peripheral ulcerative keratitis (PUK), and anterior uveitis were diagnosed in 29%, 6%, 5%, 2%, and 10%, respectively, of the mentioned cohort. Ocular ADRs to non-steroidal anti-inflammatory drugs are rarely reported and include subconjunctival hemorrhages and hemorrhagic retinopathy. In patients taking indomethacin, whorl-like corneal deposits and pigmentary retinopathy have been observed. Glucocorticoids are frequently responsible for posterior subcapsular cataracts and open-angle glaucoma. Methotrexate, the prototype of disease-modifying antirheumatic drugs (DMARDs), has been associated with the onset of ischemic optic neuropathy, retinal cotton-wool spots, and orbital non-Hodgkin’s lymphoma. Mild cystoid macular edema and punctate keratitis in patients treated with leflunomide have been occasionally reported. The most frequently occurring ADR of hydroxychloroquine is vortex keratopathy, which may progress to “bull’s eye” maculopathy. Patients taking tofacitinib, a synthetic DMARD, more frequently suffer herpes zoster virus (HZV) reactivation, including ophthalmic HZ. Tumor necrosis factor inhibitors have been associated with the paradoxical onset or recurrence of uveitis or sarcoidosis, as well as optic neuritis, demyelinating optic neuropathy, chiasmopathy, and oculomotor palsy. Recurrent episodes of PUK, multiple cotton-wool spots, and retinal hemorrhages have occasionally been reported in patients given tocilizumab, that may also be associated with HZV reactivation, possibly involving the eye. Finally, rituximab, an anti-CD20 monoclonal antibody, has rarely been associated with necrotizing scleritis, macular edema, and visual impairment.

**Conclusion:**

The level of evidence for most of the drug reactions described herein is restricted to the “likely” or “possible” rather than to the “certain” category. However, the lack of biomarkers indicative of the potential risk of ocular ADRs hinders their prevention and emphasizes the need for an accurate risk vs. benefit assessment of these therapies for each patient.

## Introduction

Rheumatoid arthritis (RA) is a chronic inflammatory autoimmune disease of unknown etiology that affects approximately 1% of the global population, or 5 per 1000 adults, and occurs two to three times more frequently in women than in men [1]. Among the 446 million inhabitants of the post-Brexit EU, at least 3 million suffer from RA. In an Italian study of an administrative cohort comprising 2,268,514 males and 2,446,769 females ≥ 18 years of age, the prevalence of active RA was 0.32% (95% confidence interval [CI]: 0.38–0.44), and the yearly incidence for women and men was 48 per 100,000 (95% CI: 40–57) and 20 per 100,000 (95% CI: 10–30) [2]. In addition to its significant morbidity and mortality, RA is frequently associated with severe physical disability, impacts patient’s work productivity and well-being, and imposes a major financial burden on healthcare systems and society [3].

Disease-modifying antirheumatic drugs (DMARDs) have long been considered the gold standard or cornerstone of treatment for RA, in that they are able to interfere with the signs and symptoms of the disease and prevent the progression of joint involvement [4]. The introduction of biologics [5] and the consequent possibility of timely treatment have led to clinical remission [6] or at least a condition of low disease activity (LDA), as assessed by instruments such as the Clinical Disease Activity Index (CDAI), in a growing number of patients [7]. The aim of the “treat-to-target” strategy is to improve the CDAI by ≥ 50% within 3 months and thus possibly attain remission in patients with early RA, or clinical remission or LDA within 6 months in those with established RA [7].

As expected, the expanding therapeutic armamentarium has been associated with an increase in the number and types of adverse drug reactions (ADRs) that in many cases force the patient to discontinue therapy. According to the World Health Organization (WHO), an ADR is defined “any noxious, unintended and undesired effect of a drug, which occurs at doses used in humans for prophylaxis, diagnosis, or therapy” [8]. The clinical spectrum of ADRs ranges from mild upper respiratory tract infections to more severe infectious complications and the reactivation of tuberculosis; from hypersensitivity reactions to gastrointestinal involvement; from bone marrow suppression and pancytopenia to an exacerbation of demyelinating diseases; and to the onset of malignancies [5, 9].

Although RA is by definition a polyarticular disease that involves both small and large joints, in most patients symmetric, extra-articular manifestations are not uncommon, especially when treatment is delayed or underdosed. A necrotizing vasculitis of the small and medium-sized arteries may affect several organs, including the eye [10]. In fact, the eye may be the target of both RA in the active phase and of several drugs commonly employed to treat the disease.

In this paper, we first summarize the most common and well-known ophthalmological manifestations of RA and then focus on ADRs, whether involving conventional first- or second-line drugs or subsequent forms of treatment, and their damage to the visual system. ADRs induced by antirheumatic drugs in RA patients but not involving the eye and those occurring in conditions other than RA, with or without ocular involvement, are not discussed. Our own observations collected over a period of 20 years in a cohort of 439 RA patients will be the subject of another paper.

### Search strategy

A systematic review of studies (case reports, case series, reviews, clinical trials, retrospective, and prospective studies) published in the PubMed, MEDLINE, and EMBASE databases from the beginning of 1960 up to December 2020 was performed. The search terms included ocular signs and symptoms of RA, ocular side effects or adverse events or adverse reactions, and each of the antirheumatic drugs listed in Table 1. The search was restricted, with a few exceptions, to English-language publications.

**Table 1:** Provisional list of disease-modifying antirheumatic drugs (DMARDs)

Category		Corresponding drugs
Synthetic DMARDs	Conventional synthetic (cs) DMARDs	Conventional:
		• Methotrexate
		• Leflunomide
		• Sulfasalazine
		• Hydroxychloroquine
	Targeted synthetic (ts) DMARDs	Janus kinase inhibitors:
		• Tofacitinib
		• Baricitinib
		• Filgotinib
		• Upadacitinib
Biological DMARDs	Biological originator (bo) DMARDs	TNF inhibitors:
		• Adalimumab
		• Certolizumab
		• Etanercept
		• Golimumab
		• Infliximab
		IL-6R inhibitors:
		• Tocilizumab
		• Sarilumab
	Biological (b) DMARDs	Co-stimulation inhibitors:
		• Abatacept
		Anti-CD20:
		• Rituximab
	Biosimilar (bs) DMARDs	Currently available:
		• Adalimumab
		• Etanercept
		• Infliximab
		• Rituximab

List incomplete due to the frequent advent of new drugs

### Ocular manifestations of RA

Ocular signs and symptoms of variable severity may occur in patients with long-standing RA and are sometimes the presenting features of the disease. A recent meta-analysis of ocular complications across the spectrum of rheumatic diseases determined a prevalence in RA of approximately 18% of patients, indicating that the eye is a common extra-articular target of the disease [10]. The most common ocular symptoms are grittiness, discomfort and redness, variable pain, and vision disturbances. Studies of RA that have included an ophthalmological examination have shown the more frequent involvement of the anterior segment of the eye, including keratoconjunctivitis sicca (KCS), episcleritis, scleritis, peripheral ulcerative keratitis (PUK), and anterior uveitis (AU) [11, 12], with retinal vasculitis as a rare manifestation. Reports in the literature evidence the variable incidence and prevalence of these conditions, possibly depending on environmental factors as well as the ethnic and genetic characteristics of the patients, disease duration and stage, and the appropriateness of therapy [13, 14]. Overall, it is likely that ocular manifestations of RA are overlooked and/or underdiagnosed to a significant extent [11].

**Fig. 1:**
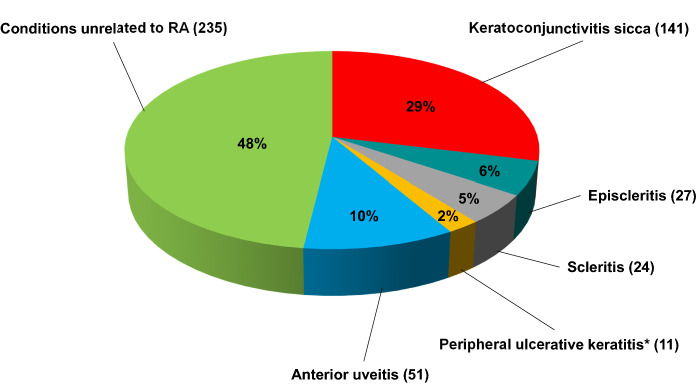
Ophthalmological diagnoses in 489 patients with rheumatoid arthritis (RA). Within this group, 52% of their conditions were strictly related to the underlying RA. *Peripheral ulcerative keratitis progressed to perforation of the cornea in the left eye of one patient and to corneal melt syndrome in the left eye of another patient

**Fig. 2:**
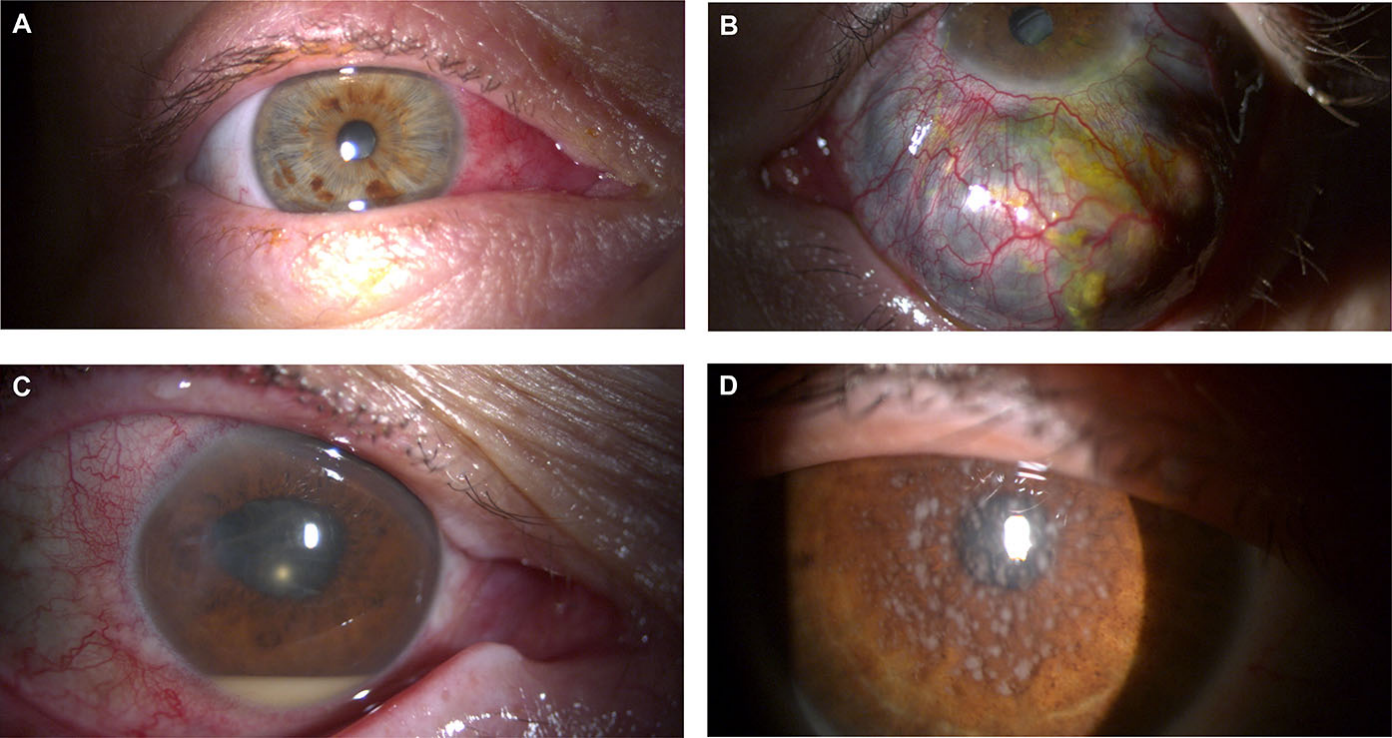
**a** Right eye of a 53-year-old male patient with rheumatoid arthritis (RA) and bilateral simple episcleritis who presented with redness of both eyes, lacrimation, photophobia, and mild discomfort. Note the congested and prominent blood vessels of the nasal episclera. He was initially treated with fluoromethalone 1% eye drops three times daily, and, because of only partial efficacy, with prednisolone acetate 1% plus oral indomethacin 100 mg daily for one week, which resulted in a gradual reduction of the inflammation. Following frequent recurrences, infliximab was added to these conventional synthetic disease-modifying antirheumatic drugs for the treatment of the underlying RA. **b** Anterior multinodular scleritis, prevalent in the left eye, complicating active RA in a 47-year-old female patient who complained of moderate ocular pain, often stabbing in character, and a fall in visual acuity. Discrete scleral nodules, displacement of the deep vessels over the nodules, and surrounding inflammation overlying the unaffected sclera can be seen. The patient received topical steroids and weekly subcutaneous injections of methotrexate (15 mg) plus a 6-week course of oral methylprednisolone (12 mg per day), followed by gradual tapering. Two months later, the multinodular scleritis had completely regressed. **c** Anterior uveitis with hypopyon in a 69-year-old female patient with severe long-standing RA under treatment with methotrexate (15 mg weekly) plus adalimumab (40 mg subcutaneously every 2 weeks). The patient initially complained of pain, decreased vision, tearing, photophobia, and eye redness without discharge. Anterior inflammation with hypopyon was detected in her left eye, and her visual acuity dropped to 4/200, but no pathogens were demonstrated in the intraocular fluid collected by vitrectomy. Following the discontinuation of methotrexate and adalimumab, the ocular inflammation gradually improved and eventually resolved with the daily oral use of amoxycillin (850 mg) plus clavulanic acid (125 mg) and prednisone (15 mg). **d** Slit-lamp photograph from a 55-year-old female patient with RA and recurrent episodes of sarcoid anterior uveitis with multiple granulomatous mutton-fat keratic precipitates. Intraocular pressure was 25 mmHg in each eye. The patient described bilateral photophobia and a fall in visual acuity beginning ~ 1 month prior to presentation. Treatment with systemic and topical steroids in addition to methotrexate resulted in a marked improvement and sustained remission

Figure 1 summarizes our observations in a cohort of 489 patients with established RA who attended the Internal Medicine and Ophthalmology departments in Bari between 1993 and 2019. The ocular manifestations in slightly more than half of the cohort were the typical manifestations of RA, whereas those of the remaining patients included a heterogeneous array of ophthalmological conditions unrelated to RA, such as hypertensive or diabetic retinopathy, cataract, infectious posterior or intermediate uveitis, allergic conjunctivitis, and strabismus. As expected, KCS and AU were the most frequent forms of ocular involvement, but in none of our RA patients did the ocular findings precede the articular manifestations of the disease. Representative examples of the distribution of the different ocular manifestations in RA are reported in Fig. 2.

### DMARDs

As RA is a disease of unknown etiology and therefore without a causally directed therapy, it remains chronic in its course. Glucocorticoids (GCs), non-steroidal anti-inflammatory drugs (NSAIDs), and pain medications relieve the symptoms of RA but are unable to prevent the progressive joint damage and eventual disability. Typically, these drugs are used either to treat the initial phase of the disease, before the diagnosis is confirmed, or as an additional symptomatic form of treatment [1]. Conventional synthetic (cs) DMARDs, by contrast, have been used in RA patients worldwide for over 50 years, given the established ability of these agents to interfere with the clinical course of the disease and prevent further joint damage. Administered alone or in combination with GCs, csDMARDs, and especially methotrexate (MTX), are able to induce remission or at least LDA in approximately 50% of patients [1].

A provisional list of cs, targeted synthetic (ts), biologic, and biosimilar (bs) DMARDs is provided in Table 1. Progressively decreasing response rates to biologic agents have been observed according to whether the study population comprises patients with early RA or established RA, is MTX-naïve or MTX-experienced, or includes patients with late anti-tumor necrosis factor (TNF)-experienced RA [15]. Among patients diagnosed with early-stage disease and administered appropriate treatment, csDMARDs and tsDMARDs are able to induce LDA or even remission in 75–80% [5].

### EULAR recommendations for the management of RA

The European League Against Rheumatism (EULAR) has developed and repeatedly updated a series of overarching principles and therapeutic recommendations, with the aim of providing rheumatologists, other specialists, health professionals, patients, and stakeholders with expert advice on the rational use of clinically effective drugs, their possible combinations, and timely therapeutic adaptation [5]. The 2019 updated EULAR recommendations for the management of RA are summarized in Table 2. In brief, treatment should be started with csDMARDs administered together with low-dose GCs. Non-responders or patients with markers of a poor prognosis (occurrence of erosions, rheumatoid factor, or anti–citrullinated protein antibodies, and a baseline Disease Activity Score in 28 joints based on C-reactive protein) should be switched to biologic therapy with the aim of achieving either remission in those with early RA, or LDA in those with established RA. In the latter, following the withdrawal of biologic therapy, a good response can be maintained, and a disease flare avoided in the majority of patients by gradually tapering the dose or increasing the interval between doses [15].

**Table 2:** The 2019 updated EULAR recommendations for the management of rheumatoid arthritis [5]

1	Therapy with DMARDs should be started as soon as the diagnosis of RA is made
2	Treatment should be aimed at reaching a target of sustained remission or low disease activity in every patient
3	Monitoring should be frequent in active disease (every 1–3 months); if there is no improvement by at most 3 months after the start of treatment or the target has not been reached by 6 months, therapy should be adjusted
4	MTX should be part of the first treatment strategy
5	In patients with a contraindication to MTX (or early intolerance), leflunomide or sulfasalazine should be considered as part of the (first) treatment strategy
6	Short-term glucocorticoids should be considered when initiating or changing csDMARDs, in different dose regimens and routes of administration, but should be tapered as rapidly as clinically feasible
7	If the treatment target is not achieved with the first csDMARD strategy, in the absence of poor prognostic factors, other csDMARDs should be considered
8	If the treatment target is not achieved with the first csDMARD strategy, when and poor prognostic factors are present, a bDMARD or a tsDMARD should be added
9	bDMARDs and tsDMARDs should be combined with a csDMARD; in patients who cannot use csDMARDs as comedication, IL-6 pathway inhibitors and tsDMARDs may have some advantages compared with other bDMARDs
10	If a bDMARD or tsDMARD has failed, treatment with another bDMARD or a tsDMARD should be considered; if one TNF inhibitor therapy has failed, patients may receive an agent with another mode of action or a second TNF inhibitor
11	If a patient is in persistent remission after having tapered glucocorticoids, one can consider tapering bDMARDs or tsDMARDs, especially if this treatment is combined with a csDMARD
12	If a patient is in persistent remission, tapering the csDMARD could be considered

### Causality in ADRs

In clinical practice, it is often difficult to establish a cause-effect relationship between a drug and the occurrence of an adverse event(s). Instead, causality is usually based on the following established criteria: (a) the reaction is well documented and frequently described; (b) withdrawal of the drug results in recovery; (c) other possible causes are reasonably excluded; (d) the severity of the reaction is directly related to the dose of the drug; (e) the adverse event is objectively evident and not simply described by the patient; (f) similar drugs induce similar effects in the same patient; and (g) rechallenge of the suspected drug is associated with recurrence of the event [16, 17]. The WHO has also developed causality assessment guidelines to categorize suspected ADRs (Table 3) [18].

**Table 3:** The World Health Organization’s causality assessment of suspected adverse drug reactions [18]

Category of adverse drug reaction	Definition
Certain	A clinical event, including laboratory test abnormality, occurring in a plausible time relationship to drug administration, and which cannot be explained by concurrent disease or other drugs or chemicals. The response to withdrawal of the drug (dechallenge) should be clinically plausible. The event must be definitive pharmacologically or phenomenologically, using a satisfactory rechallenge procedure if necessary
Probable/likely	A clinical event, including laboratory test abnormality, with a reasonable time sequence to administration of the drug, unlikely to be attributed to concurrent disease or other drugs or chemicals, and which follows a clinically reasonable response on withdrawal (dechallenge). Rechallenge information is not required to fulfill this definition
Possible	A clinical event, including laboratory test abnormality, with a reasonable time sequence to administration of the drug, but which could also be explained by concurrent disease or other drugs or chemicals. Information on drug withdrawal may be lacking or unclear
Unlikely	A clinical event, including laboratory test abnormality, with a temporal relationship to drug administration that makes a causal relationship improbable, and in which other drugs, chemicals, or underlying disease provide plausible explanations
Conditional/unclassified	A clinical event, including laboratory test abnormality, reported as an adverse reaction, about which more data are essential for a proper assessment or the additional data are under examination
Unassessable/unclassifiable	A report suggesting an adverse reaction that cannot be judged because information is insufficient or contradictory, and which cannot be supplemented or verified

### Ocular ADR to NSAIDs

The role of NSAIDs in the treatment of RA is now marginal, because their action is limited to mitigating the symptomatology, due to their analgesic effect, whereas they are unable to prevent or arrest the progression of joint damage and related disability. Therefore, as noted above, NSAIDs are commonly used in the initial phases, when the diagnosis of RA has not been fully established, or as adjunctive symptomatic therapy. In addition to inducing gastrointestinal, cardiovascular, and renal adverse reactions, the NSAIDs piroxicam, ibuprofen, and naproxen as well as acetylsalicylic acid may be associated with an increased bleeding tendency, especially when administered for long periods of time and at high doses. Under those conditions, subconjunctival hemorrhages and hemorrhagic retinopathy, subjectively reported by the patient as photophobia and blurred vision, have been described, albeit rarely [19–21].

The long-term administration of indomethacin has been shown to lead to corneal opacities, with patients complaining of blurred vision and photophobia [22, 23]. In a few patients who have been taking indomethacin continuously for two or more years, whorl-like corneal deposits have been observed, somewhat reminiscent, on slit-lamp examination, of the *cornea verticillata* pattern characteristic of hydroxychloroquine (HCQ) keratopathy [24]. When the drug is discontinued, the corneal changes may slowly fade or, more rarely, disappear. Indomethacin has also been associated with retinopathy, characterized by pigmentary changes of the macula and in some cases scattering of the retinal pigment epithelium near the fovea, with or without the development of multiple small areas of depigmentation around the macula [24–26].

### Ocular ADR to GCs

Despite the availability of csDMARDs, GCs are still co-prescribed to control inflammation in early-stage RA but are then gradually tapered until their discontinuation within a few months, at which time a treat-to-target strategy is pursued [1]. The longest duration of GC use occurs in COBRA combination therapy, a step‐down DMARD strategy consisting of an oral pulse of prednisolone progressively tapered from 60 mg/day to complete withdrawal at week 28, associated with the administration of low‐dose MTX (7.5 mg/week) for 40 weeks and a maintenance dosage of sulfasalazine (2 g/day) [27]. EULAR, instead, recommends that newly diagnosed patients be treated with MTX (7.5–15 mg/week) combined with oral GCs at a dose of up to 30 mg prednisone equivalents/day, tapered to 0 over 3–4 months [28].

However, a recent UK study on the changes in the pharmacological management of 71,411 RA patients over two decades showed that, contrary to the most recent recommendations on RA treatment, GC use post-diagnosis remains substantial both at 3 years (18%) and at 15 years (17%) [29]. It is therefore not surprising that, in addition to the well-known side effects of GCs, such as infections, activation of latent tuberculosis, type 2 diabetes mellitus, peptic ulcer, and osteoporosis, several types of ocular ADRs may develop in RA patients. The cumulative and average daily GC doses are directly related to the risk of developing posterior subcapsular cataracts (PSCs), although with large variations between patients [24, 30]. A daily prednisone-equivalent dose of ≥ 10 mg for longer than one year leads to the onset of cataracts in approximately 75% of patients [31], but even a daily dose of 5 mg for 2 months may result in the onset of PSC among those who are susceptible [32]. Drug withdrawal rarely, if ever, includes regression of the lens opacities.

A persistent increase in intraocular pressure (IOP) leads to the onset of open-angle glaucoma with variable frequency, again depending on the patient’s susceptibility and the frequency and dose of GCs [24]. The disease process, underpinned by progressive degeneration and cupping of the optic disc, is insidious and deceptive, in that it initially affects the peripheral vision but then slowly spreads toward the center of the eye, ending in a loss of the visual field. Consequently, most patients remain asymptomatic or poorly symptomatic and undiagnosed until the disease is in an advanced stage. In a subset of patients, open-angle glaucoma is linked to genetic factors, namely, a mutation in the myocilin gene that is inherited in an autosomal dominant fashion and is characterized by frequent nucleotide substitutions [33, 34]. A database on myocilin genotypes may help clinicians and researchers to identify individuals at risk of developing open-angle glaucoma [35].

In patients with an increased IOP, GCs should be tapered or discontinued, although the time necessary to achieve a pressure reduction is roughly related to the length of GC use. Some patients will require medical or surgical procedures to effectively lower the IOP [36]. The treatment of steroid-induced glaucoma is largely similar to that of primary open-angle glaucoma and includes beta-blockers, alpha-2 agonists, and carbonic anhydrase inhibitors. For patients who do not tolerate anti-glaucoma agents or who are unresponsive to medical management, laser trabeculoplasty is usually advised, especially if optic nerve damage is impending. The third therapeutic alternative, trabeculectomy, is reserved for patients recalcitrant to medical and laser treatments or likely to be further treated with GCs.

### Ocular ADR to csDMARDs

In the following, a short overview of the ocular side effects associated with the drugs commonly employed for the treatment of RA and listed in Table 1 is provided.

#### MTX

In addition to pancytopenia, myelosuppression, and hepatic and pulmonary toxicity, the potential induction of ocular toxicity by the folic acid antagonist MTX is of particular importance, given that MTX is the “anchor drug”, a major therapeutic weapon in the treatment of RA, and therefore the most frequently employed csDMARD.

Non-arteritic ischemic optic neuropathy was described at the beginning of the 2000s, before a relationship between folic acid and MTX was determined [37, 38]. Since then and based on the demonstration that MTX increases plasma homocysteine levels, patients treated with MTX are given either folic or folinic acid supplements to decrease plasma homocysteine levels and prevent organ damage, including optic neuropathy. The change in the homocysteine level is apparently unrelated to the presence or absence of the C677T mutation in the MTHFR gene [39].

A variable combination of ocular pain, itching, photophobia, blurred vision, periorbital edema, blepharitis, and conjunctivitis is observed in 6–10% of RA patients receiving subcutaneous injections of MTX at a dose of 15–25 mg/week. These ocular signs typically develop during the first 3 to 5 administrations and then gradually fade or disappear [40].

Retinal cotton-wool spots are a rare finding and were detected in both eyes in a woman with RA who had been treated with MTX for 11 years and whose laboratory examinations revealed severe pancytopenia. Tapering of the drug resulted in regression of the cotton-wool spots [41]. This observation suggests that MTX can also induce ischemic retinal complications, a finding that should also raise suspicion of bone marrow suppression and pancytopenia.

A two-fold increased risk of both Hodgkin's lymphoma (HL) and non-Hodgkin's lymphoma (NHL), with or without ocular involvement, has been reported in RA patients, with diffuse large B cell NHL reported most frequently [42]. Whether treatment with MTX or other immunosuppressive agents affects the occurrence and subtype of lymphoma has not been established, but large cohort studies seem to rule out a role for either MTX or tumor necrosis factor inhibitors (TNFis; discussed below) in increasing the lymphoma risk [42, 43]. Instead, RA disease activity and the consequent persistent immunologic stimulation may in themselves increase the risk of lymphoma, such that MTX and TNFis, by reducing the aggressiveness of RA, may indirectly reduce the risk of later complications such as lymphoma [43]. However, a case in which a 78-year-old woman with RA developed orbital mucosa-associated lymphoid tissue (MALT) NHL was reported. The patient had been treated with MTX for over 8 years when she complained of swelling of the left upper eyelid. Interestingly, the subconjunctival and orbital masses regressed within 10 months after MTX withdrawal and did not recur over the next 2 years, thus supporting the authors’ conclusion that this was a case of MTX-induced MALT lymphoma [44].

#### Leflunomide

Leflunomide, a dihydroorotate dehydrogenase inhibitor and immunomodulatory agent, is known mostly for its hepatotoxicity, whereas ocular side effects have been rarely reported. However, 2 weeks after the initiation of leflunomide treatment, a 57-year-old male patient with RA developed blurred vision in both eyes. Funduscopic examination and fluorescein angiography revealed mild cystoid macular edema in the right eye and mild but more prominent cystoid macular edema in the left eye. Three months after leflunomide discontinuation, his visual acuity returned to normal, without evidence of cystoid macular edema on the clinical examination [45].

A woman with RA refractory to sulfasalazine and MTX was switched to leflunomide. Two weeks later, she developed a rapidly spreading maculopapular rash as well as ulcers of the ocular and oral mucosa. Following leflunomide discontinuation, the skin lesions healed over the next month, but punctate keratitis with keratinization of the cornea caused a complete loss of vision [46].

#### Sulfasalazine

The sulfonamide sulfasalazine is a prodrug that consists of two therapeutic compounds coupled via an azo linkage: sulfapyridine, with anti-bacterial activity, and 5-amino-salicylic acid, with anti-inflammatory properties. Taken orally, sulfasalazine is broken down by the intestinal flora and its bioactive components released into the blood.

Liver injury is the main toxic effect of sulfasalazine whereas ocular side effects have rarely been reported, although the drug has been used for many years in the treatment of RA. A young female patient who was a contact lens wearer complained of a sudden increase in myopia 3 weeks after the NSAID she had long been taking, meloxicam, was combined with sulfasalazine. The discontinuation of sulfasalazine resulted in the improvement of myopia and in stable visual acuity [47].

#### Hydroxychloroquine

HCQ, an analog of chloroquine, is an antimalarial agent used in the treatment of RA and other autoimmune disorders. Ocular toxicity related to HCQ use includes keratopathy, lens opacities, ciliary body dysfunction, retinal damage, and pigmentary retinopathy [24]. Our group previously described the potential risks for ocular adverse events, including vortex keratopathy and vision-destroying maculopathy, in patients with systemic lupus erythematosus that has long been treated with HCQ [48, 49]. HCQ retinal toxicity is more frequent than commonly thought, as an overall prevalence of 7.5% has been reported in patients on HCQ for > 5 years, rising to almost 20% after 20 years of treatment [50]. In addition to the length of administration, a daily dose > 6.5 mg/kg, a high cumulative dose, and co-existing renal disease are risk factors of HCQ retinopathy [51].

The most frequently occurring ocular manifestation of HCQ toxicity is vortex keratopathy (VK), also referred to as *cornea verticillata* because of the whorl-like corneal deposits. Other possible causes of VK are amiodarone, ibuprofen, or tamoxifen use and the lysosomal storage disorder Fabry’s disease [52, 53]. A less frequent but more ominous manifestation of HCQ toxicity is “bull’s eye” maculopathy, whose early stage is not associated with detectable anatomic abnormalities and is therefore asymptomatic, whereas in the more advanced stage, fundus examination and a fundus autofluorescence scan usually reveal an abnormal pigmentation over the macula, consisting of a central hyperpigmentation surrounded by a hypopigmented ring, thus recalling a bull's eye target. In patients who develop VK or maculopathy, HCQ must be discontinued immediately. While this usually results in a slow reduction and eventual regression of the subjective symptoms and corneal deposits in those with VK, toxicity can still progress in patients with maculopathy, given that the half-life of HCQ is one month or longer, and a period of 6 months may be required for a full washout of the drug [49, 54].

According to the American Academy of Ophthalmology Statement, in addition to a baseline eye examination to rule out pre-existing macular disease, patients receiving HCQ for 5 years or longer should undergo annual eye-care visits that include automated visual fields and spectral domain-ocular coherence tomography as primary screening tools, and fundus fluorescein angiography and conventional full-field or multifocal electroretinography as additional screening instruments [52]. The prompt detection of toxic effects and immediate drug discontinuation usually result in visual improvement of variable extent, whereas a late diagnosis is inevitably followed by visual deterioration.

### Ocular ADR to tsDMARDs

The Janus kinase–signal transducers and activators of transcription (JAK-STAT) pathway plays a crucial role in the pathogenesis of RA. The cytokines released in RA bind to the type I/II cytokine receptor family, which employs the JAK-STAT pathway to effect signal transduction. Following the binding of a type I/II cytokine to its cognate receptor, receptor-associated JAKs are activated and phosphorylate STATs, resulting in the activation of cytokine-specific genetic programs [55, 56]. Based on this model, orally bioavailable JAK inhibitors have been introduced as a class of synthetic targeted drugs that includes a growing number of molecules (Table 2). In patients with poor prognostic factors and in whom MTX or other csDMARDs are unable to achieve the treatment target, the addition of JAK inhibitors may result in better disease control [15].

Tofacitinib was introduced in 2014 as a new-in-class JAK inhibitor for the treatment of RA, with the subsequent approval of other low-molecular-weight compounds of the same class. Although worldwide experience with these drugs is thus far limited, the reported adverse events, almost exclusively involving tofacitinib, include an increased risk of heart problems, cancer, pancytopenia, dyslipidemia, increased liver enzymes, cardiovascular events, venous thromboembolism, lower respiratory tract infections, and herpes zoster (HZ) virus reactivation [57].

A careful search of the literature for ocular adverse events associated with JAK inhibitors revealed a study of 4789 RA patients, 239 (5%) of whom developed tofacitinib-associated HZ, including 2 patients with ophthalmic HZ (0.8%) [58]. Six additional patients with ophthalmic HZ were described in a comprehensive review [59]. The major difference in the adverse events associated with tofacitinib compared with other csDMARDs and bDMARDs is the striking increase in the occurrence of HZ, especially in Asia. The baseline risk of HZ among RA patients is two to threefold higher than in the general population [60]. The incidence of HZ among patients receiving tofacitinib ranges from 2.1/100 patient-years for patients receiving a dose of 5 mg b.i.d. to 8.6/100 patient-years for patients treated with a dose of 10 mg b.i.d. [59]. Intravenous followed by oral valacyclovir is usually able to induce a progressive improvement of shingles, but the reduction in visual acuity is a long-lasting complication. Post-herpetic neuralgia can persist for 2–3 months or longer.

#### Ocular ADR to TNFis

TNF has been implicated in joint destruction and synovial hyperplasia in RA patients [61–63], such that TNFis, a subset of bDMARDs, have been effective in dramatically changing the therapeutic landscape of the disease. Currently, TNFis include adalimumab, golimumab, and infliximab, which are TNF-specific monoclonal antibodies (mAbs); certolizumab, a TNF-specific Fab fragment bound to polyethylene glycol; and etanercept, a fusion protein comprising two TNF receptor-2 extracellular domains fused to a single human IgG1 Fc fragment (Table 2) [62]. These five TNFis show equivalent efficacy.

TNFis are frequently employed as first- or second-line GC-sparing agents for patients with non-infectious uveitis [64] but, paradoxically, in some patients their administration may result in the onset or recurrence of inflammatory eye disease. Uveitis was reported in association with the use of TNFis in 20 patients treated with etanercept, in 4 treated with infliximab, and in 2 treated with adalimumab [65]. After adjustment for the different number of patients taking each medication, uveitis cases associated with etanercept were significantly more frequent than those associated with infliximab and adalimumab. It should be noted that the onset of uveitis was independent of the length of TNFi exposure, and that in at least 2 patients dechallenge/rechallenge with etanercept led to the resolution of uveitis when the drug was stopped and to relapse when it was reintroduced [65].

A review of the literature that included papers published until the end of 2020 yielded 85 cases of TNFi-induced sarcoidosis, with or without ocular involvement. Thirty-eight of the patients had been diagnosed with RA (44.7%). The mean length of TNFi administration before the onset of sarcoidosis was 18 months (range 1–84 months). Discontinuation of the TNFis in 71 patients resulted in the regression of sarcoidosis in 36 patients and stable disease in 2. In the remaining 33 patients, sarcoidosis resolved in 32 following systemic GC treatment, but persisted in 1 patient [66].

Ocular manifestations of sarcoidosis, isolated or accompanied by the involvement of other organs, have been detected in several patients [67–80], as summarized in Table 4. Of particular interest are the cases, independently reported, of two female RA patients (54- and 59-years-old) who developed sarcoid-like disease during etanercept treatment. In both, the drug was withdrawn, and the patients’ condition partially improved [72, 81]. A switch to adalimumab resulted in complete recovery and in the second patient included the regression of sarcoid granulomatous uveitis (corner nodules and snowball opacities) [72]. The pathogenetic pathway of sarcoidosis is still poorly defined, but a Th1-like cytokine pattern characterized by increased levels of TNF-α, IFN-γ, and T-cell responses may play an important role [82]. In this context, it is important to emphasize that etanercept and adalimumab are both TNFis, but one is a soluble dimeric fusion protein that mimics native TNF receptors, while the other is a fully humanized recombinant IgG1 anti-TNF-α mAb. Thus, it is likely that the observed discrepancies were related to the different immunological effects, in that etanercept (but not adalimumab) increases the production of IFN-γ whereas adalimumab (but not etanercept) induces the lysis of T-cells and TNF-α-expressing monocytes [72, 81, 83]. However, the opposite has also been described, as a patient with ankylosing spondylitis and adalimumab-induced sarcoidosis who was switched to etanercept had no recurrence of pulmonary sarcoidosis, suggesting that etanercept is a treatment option for patients who develop paradoxical sarcoid-like reactions in response to adalimumab [84].

**Table 4:** Literature search for ocular adverse reactions (sarcoid-like and tubercular granulomatous uveitis) to TNFis in patients with established RA

References	Sex, age (years)	Duration of anti-TNF therapy (months) at symptoms onset	Ocular findings	Diagnosis/treatment/outcome
[67]	F, 41	Infliximab (68)	Diplopia and nerve palsy of the left eye, with severe papilledema in both eyes	Bilateral granulomatous iridocyclitis and retinal periphlebitis typical for sarcoidosis. Neurosarcoidosis with papilledema. Infliximab was discontinued and high-dose GCs plus MTX were given. After a ventriculoperitoneal shunt, the papilledema and iridocyclitis regressed
[68]	F, 51	Etanercept (5)	Bilateral ocular pain, increased intraocular pressure, multiple nodules and peripheral synechiae on the trabecular meshwork, with focal chorioretinal exudates and retinal periphlebitis	Recurrent iridocyclitis and multiple nodules on the trabecular meshwork in both eyes. Systemic sarcoidosis was diagnosed. The withdrawal of etanercept and a brief course of GCs led to the control of the patient’s uveitis
[69]	F, 69	Etanercept (27)	Bilateral anterior uveitis	Diagnosis of sarcoid-like granulomatosis. Following a reduction in the GC dose, both the uveitis and the cutaneous and pulmonary features relapsed. The replacement of etanercept by adalimumab led to resolution within a few weeks
[70]	F, 49	Infliximab (60)	Intermittent red, painful eyes	Acute bilateral anterior uveitis was diagnosed in the context of multisystem sarcoidosis. Infliximab discontinuation and treatment with GCs resulted in a general improvement
[71]	F, 61	Adalimumab (48)	Bilateral panuveitis with venous vasculitis and peripheral multifocal choroiditis	A biopsy of a papular lesion on the forehead showed noncaseating granulomas compatible with sarcoidosis. The replacement of adalimumab with GC therapy led to the resolution of skin involvement and an improvement of the panuveitis
[72]	F, 54	Etanercept (undefined)	Bilateral panuveitis	Sarcoid uveitis was diagnosed. The anterior uveitis resolved completely and the posterior uveitis partially after etanercept termination,. Ten months later, she was started on adalimumab, with complete recovery
[73]	F, 40	Etanercept (84)	Anterior uveitis, bilateral Bell’s phenomenon, and left papilledema	After a diagnosis of severe neurosarcoidosis, etanercept was stopped and GCs were initiated. One year later, she remained on MTX and GCs. Her eyesight completely recovered but not the facial paralysis
[74]	F, 64	Certolizumab (36)	Bilateral uveitis with mild flare in the anterior chambers and posterior synechiae. Vitreal cells and haze, more prominent in the left eye. Macular edema and peripheral retinal punched-out lesions	General features suggestive of sarcoidosis. Topical steroid drops resulted in an improvement of the uveitis. Certolizumab was discontinued and MTX was increased. Two months later, her uveitis had decreased but the macular edema persisted
[75]	F, 33	Etanercept (undefined) Previous trials of adalimumab and infliximab	Bilateral refractory uveitis	Neurological signs ascribed to neurosarcoidosis. Etanercept was withdrawn and the patient was treated with GCs in addition to MTX and infliximab, with clinical improvement
[76]	F, 54	Etanercept (6)	Bilateral iritis with congestion of the bulbar conjunctiva	Lymph node biopsy consistent with sarcoidosis. After etanercept treatment was stopped, a follow-up examination showed improvement without therapeutic intervention
[77]	F, 68	Etanercept (72)	Unilateral anterior uveitis	Etanercept was maintained and resolution was achieved with topical GCs
[77]	M, 71	Infliximab (undefined)	Unilateral uveitis with massive granulomatous keratic precipitates	Infliximab was discontinued. Certolizumab, oral GCs, and MTX induced complete recovery
[78]	F, 40	Etanercept (32)	Bilateral acute anterior uveitis	Steroid drops yielded a favorable response. Etanercept was maintained
[78]	F, 64	Etanercept (38)	Acute anterior granulomatous uveitis	Etanercept discontinued for 12 months. Uveitis was treated locally, with a favorable response, but disease relapse occurred 5 months after etanercept reintroduction
[78]	F, 28	Etanercept (1) after Infliximab	Acute anterior uveitis	Uveitis resolved with local GC drops but disease relapse occurred 3 times. Adalimumab was introduced
[78]	F, 65	Etanercept (2) after infliximab	Acute anterior hyalitis and macular edema	Systemic GCs yielded a favorable response. Etanercept was continued but after 18 months it was replaced by adalimumab, without relapse
[78]	M, 64	Infliximab (10)	Acute anterior uveitis and detachment of the retina	Successful local treatment of uveitis while continuing Infliximab. Retinal reattachment surgery
[78]	F, 70	Etanercept (20)	Anterior and posterior uveitis	Etanercept was withdrawn, replaced by systemic GCs and then adalimumab
[79]	F, 48	Etanercept (18)	Left eye scleritis	The patient was given local and systemic GCs, local cyclosporine, and azathioprine. The scleritis resolved and did not recur. Etanercept was continued regularly
[79]	F, 58	Etanercept (20)	Severe anterior uveitis	Uveitis resolved and did not recur following local and systemic GCs. Etanercept was continued regularly
[80]	F, 58	Etanercept (24)	Chronic unilateral granulomatous panuveitis	After a diagnosis of tuberculous uveitis, etanercept was stopped and the patient was treated with anti-TB drugs. Four months later, the panuveitis had resolved

TNFis, tumor necrosis factor inhibitors; RA, rheumatoid arthritis; GCs, glucocorticoids; MTX, methotrexate

A recent multicenter, retrospective study investigating drug-induced sarcoid uveitis identified 16 patients, including 3 with RA that had been treated with abatacept, etanercept, and certolizumab, respectively [77].

An important point is that TNF neutralization by TNFis during chronic latent tuberculosis may result in bacterial replication within the granuloma. A possible complication in these cases is that, in addition to pulmonary and extrapulmonary involvement, the reactivation of tuberculosis may also affect the eye. Etanercept-induced unilateral tuberculous panuveitis has in fact been reported [80] (Table 4).

In step with the widespread use of TNFis in the management of RA, a variety and increasing number of additional ocular side effects that may affect all ocular structures have been described. Typical examples are anterior optic neuritis (ON), retrobulbar ON, demyelinating optic neuropathy, chiasmopathy, and oculomotor palsy as well as severe infections, retinal vein occlusion, and ocular malignancy [85–98]. Table 5 provides a short description of the ocular adverse events that have been more clearly defined.

**Table 5:** Literature search for reports of ocular adverse reactions (optic neuropathy, periorbital infection, orbital myositis, and choroidal melanoma) to TNFis in patients with established RA

References	Sex, age (years)	Anti-TNF (months of therapy at symptoms onset)	Ocular findings	Diagnosis/treatment/outcome
[83]	F, 55	Infliximab (13)	Decreased vision in the left eye associated with pain on eye movement	Retrobulbar optic neuritis was diagnosed. She was treated with GCs for 13 days. Three weeks later, her vision slowly improved and her visual field deficit resolved
[84]	M, 54	Infliximab (3)	Blurred vision and severe disk swelling. Capillary dilation and vascular leakage in both optic nerve heads on angiography	Following the diagnosis of anterior optic neuritis, the patient was treated with GCs, but his vision did not recover
[84]	F, 62	Infliximab (3)	Blurred vision. Bilateral dilation of the capillaries of the optic nerve head with profuse vascular leakage. A central scotoma in the left eye	The patient was diagnosed with anterior optic neuropathy and treated with GCs, but her vision failed to improve
[84]	M, 54	Infliximab (2)	Loss of the visual field of the right eye. Disk swelling in both eyes, with capillary dilation and vascular leakage in the optic nerve heads. A large ceco-central scotoma in the right eye	Anterior optic neuritis was diagnosed and GCs were given, but 2 months later the optic nerve head turned pale, and no improvement was observed
[85]	F, 45	Infliximab (11)	Acute monocular blurring of vision, disk swelling with capillary dilation and leakage in the optic nerve head	Infliximab-associated retrobulbar optic neuritis was diagnosed. Cessation of the TNFi and systemic GC administration resulted in favorable outcome
[86]	F, 31	Etanercept (2), switched to Infliximab (4)	Impaired visual field and left eye pain with ocular movement	Work-up highly suggestive of optic neuritis. Infliximab was terminated, and pulse followed by oral GCs were administered, with resolution of visual field defects
[87]	M, 55	Etanercept (3) plus isoniazid	Progressively worsening blurred vision in the left eye	Clinical course consistent with bilateral optic neuritis. Etanercept and isoniazid were stopped. GC administration resulted in a minor improvement in left eye visual acuity
[88]	M, 40	Adalimumab (12)	Progressive visual loss in the right eye associated with pain on movement, a dense central scotoma, and a mild nasal optic disk swelling	A diagnosis of demyelinating optic neuritis was considered. The patient’s vision gradually recovered spontaneously, although he remained on adalimumab
[89]	F, 21	Etanercept (36)	Pain and decreased vision in the right eye. A 1 + relative afferent papillary defect on the right. Edematous disks	With a diagnosis of demyelinating optic neuropathy, a course of high-dose GCs was initiated, and etanercept was discontinued, but the RA symptoms worsened
[90]	F, 63	Infliximab (5)	Near and distance blurred vision in both eyes. Visual fields showed decreased foveal sensitivity and bitemporal hemianopic scotomas	Infliximab-associated chiasmopathy was diagnosed. Following drug discontinuation, the patient experienced substantial improvement in her visual acuity and visual field
[91]	M, 47	Infliximab (15)	Painless ptosis of the right upper eyelid along with double vision in left and upgaze, with limited elevation and adduction of the right eye	The transient and isolated nature of the oculomotor palsy suggested demyelination. After infliximab was withdrawn, the diplopia and ptosis gradually resolved
[92]	F, 57	Adalimumab (36)	Right-sided dacryocystitis that developed into orbital cellulitis with a tense eyelid, blistering, and necrosis	Necrotizing periorbital infection by *Streptococcus pyogenes* was identified. Adalimumab was discontinued and antimicrobial therapy was started, followed 4 months later by etanercept
[93]	F, 42	Etanercept (5)	Diplopia, progressive eye movement limitation in any direction, and proptosis	Following a diagnosis of orbital myositis, etanercept was withdrawn. Pulsed and oral GCs plus i.v. immunoglobulins led to a normalization of eye motility
[94]	F, 61	Etanercept (72)	Acute vertical binocular diplopia and paralysis of the fourth nerve of the left eye	Acute myositis of the left inferior rectus muscle was diagnosed. Etanercept was stopped and GCs were started. RTX was added 6 months later because of RA flare-up. Ocular myositis did not relapse
[95]	M, 67	Adalimumab (16)	Bilateral diffuse scleritis with choroidal thickening. A progressively growing, pigmented choroidal mass in the left eye was followed-up	Enucleation was inevitable and revealed a ciliochoroidal melanoma
[96]	F, 53	Infliximab (12)	Low central visual acuity and impairment of the lower visual field of the right eye that occurred 4 h after an i.v. infusion of infliximab	Branch occlusion of the superior temporal vein of the right eye associated with macular cystoid edema was diagnosed. Infliximab was replaced by etanercept. Four months later, neovascularization of the retina and reduction of the macular edema were detected

The risk of developing uveal melanoma was pointed out in a Swedish population-based cohort study [99], in which the relative risk of developing invasive melanoma was 50% higher in RA patients treated with TNFis than in either RA patients not treated with biological drugs or in the general population. This is especially important for patients previously diagnosed with choroidal nevi or chronic diffuse scleritis, as reported by Damento et al. [97] (Table 5). Of the 3 patients with uveal melanoma that occurred after treatment with TNFis, one had a long history of RA. He had been treated with etanercept and, after developing a T-cell leukemia, had undergone chemotherapy that resulted in complete remission of the hematological disorder. Two years later, he developed diffuse scleritis with choroidal thickening secondary to RA, which was treated with prednisone and with leflunomide that was then switched to adalimumab. After 16 months, a pigmented choroidal mass was detected in his left eye that continued to grow and eventually led to enucleation. A ciliochoroidal melanoma was diagnosed histologically [97].

These observations further suggest a correlation between the use of TNFis and the onset of malignant tumors, as a consequence of the inhibitory effect exerted by these drugs on the immune system’s control over choroidal nevi, which are well-known risk factors for the development of malignant melanoma [97, 100]. A tumor-promoting effect may also be envisaged for chronic inflammatory processes [101], such as the diffuse scleritis diagnosed in the above-mentioned patient, who was given adalimumab after having been treated with immunosuppressive agents for a previous diagnosis of T-cell leukemia.

The obvious implications of this study are that an eye examination is advisable for all patients who will be treated with a regimen that includes TNFis and that those detected with a choroidal nevus should be examined by an ophthalmologist at regular intervals.

#### Ocular ADR to interleukin-6 receptor inhibitors (IL-6Ris)

IL-6 plays a critical role in mediating the inflammation and systemic features characteristic of RA. Thus far, two IL-6Ris, biologic drugs specific for the IL-6 pathway and targeting soluble and membrane-bound IL6Rs, have been licensed for use in RA: the humanized mAb tocilizumab (TCZ) and the fully human mAb sarilumab. Both are widely used in patients with an inadequate response to one or more bDMARDs, but they have also occasionally been administered as first-line biologics [102]. Since TCZ has a longer therapeutic history, reports of potential ocular adverse events in patients receiving IL-6Ris are confined almost exclusively to this agent.

A 65-year-old man with erosive RA that had become refractory to csDMARDs was started on TCZ and responded favorably to therapy. After 11 months, he experienced an episode of PUK, followed by two additional episodes of keratitis when TCZ was reintroduced after a delay of 1 month and then 2 months. The drug was therefore discontinued [78]. Thus, similar to anti-TNFis, a paradoxical effect may sometimes be observed with IL-6Ris.

Serious ophthalmological adverse events were described in a 43-year-old woman with a 9-year history of RA that had become refractory to MTX. She was given a first intravenous infusion of TCZ, but 20 days later developed skin eruptions on her palms, soles of the feet, and in the lumbar region. Following a second TCZ infusion, a skin ulcer appeared on the right external malleolus; 4 weeks later fundus examination showed bilateral multiple cotton-wool spots and retinal hemorrhages around the optic disc. TCZ was discontinued, and the patient was placed on antibiotics and GCs, which resulted in the gradual improvement of the skin manifestations and resolution of the retinal hemorrhages, but the cotton-wool spots persisted. The TCZ-mediated inhibition of IL-6 binding to IL-6R may have caused a large increase in the patient’s serum IL-6 levels that in turn enhanced blood coagulation or favored an immune complex-mediated vasculitis by impairing the retinal microcirculation [103].

As noted above, administration of the JAK inhibitor tofacitinib may be associated with a reactivation of latent varicella zoster virus (VZV) and the appearance of HZ, possibly involving the eye. The same may be true of TCZ. A 64-year-old woman with RA had been treated with csDMARDs and TNFis, but with only transient benefit. She was then switched to TCZ and MTX, which had favorable clinical and biological effects. After 9 months of TCZ administration, she developed HZ duplex bilateralis, consisting of a right ophthalmic HZ and a C4 metamere HZ. The involvement of two non-contiguous dermatomes and both sides of the body was a remarkably rare manifestation of HZ. Local treatment in combination with valacyclovir resulted in significant improvement but, following each injection of a lower dose of TCZ, she experienced a relapse of HZ. Ten months later, TCZ was successfully replaced by rituximab (RTX) [104].

The rare adverse events that occur in RA patients treated with sarilumab should also be mentioned. A worsening of the disease and the development of retinal infiltrates were described in 8% and 5%, respectively, of a cohort of patients with posterior segment non-infectious uveitis [105].

#### Ocular ADR to co-stimulation inhibitors

T-cells contribute to the pathogenesis of RA [106], as the clinical signs and symptoms of the disease are in fact modulated by an interaction between CD28 and CD80/CD86. In particular, T-cell activation requires both an antigen-specific signal, derived from T-cell receptor recognition of antigens presented in the context of MHC molecules on the surface of antigen-presenting cells, and a second co-stimulatory signal. Abatacept, a fusion protein containing components of IgG and cytotoxic T-lymphocyte-associated protein 4, was developed to interrupt the CD28–CD80/CD86 interaction and prevent T-cell activation. The drug has been clinically effective in RA patients with an inadequate response to TNFis [107].

In a study carried out on 32 patients that evaluated the efficacy and safety of abatacept for secondary Sjögren's syndrome associated with RA, the only ocular adverse event was an infected corneal ulceration [108].

#### Ocular ADR to anti-CD20 mAbs

RTX, the first-in-class of the anti-CD20 mAbs, selectively targets B cells and is extensively used in the treatment of B cell lymphoproliferative disorders as well as a variety of autoimmune diseases. It has also been employed as a therapeutic alternative in patients with rheumatoid vasculitis refractory to non-biologic DMARDs and/or anti-TNF therapy [109].

Like other immunosuppressive agents, RTX is associated with an increased risk of opportunistic infections and the reactivation of latent virus infections. This was the case in a 47-year-old woman with RA who at the age of 36 developed bilateral recurrent nodular scleritis that became necrotizing in her right eye and progressed to macular edema with visual impairment. Over the following years, she was treated with GCs, adalimumab, cyclophosphamide, and finally with RTX. After the fourth cycle of RTX, she complained of vision loss in her right eye. Fundus examination showed hemorrhagic occlusive vasculitis, such that the differential diagnosis included acute retinal necrosis and cytomegalovirus retinitis. RTX was immediately discontinued, replaced by ganciclovir. Examination of her anterior chamber fluid showed positivity for herpes simplex virus type I. Following a switch to intravenous acyclovir plus GCs, there was no further progression. At her last follow-up, she had stable scleromalacia, defined as necrotizing scleritis in the absence of clinical inflammation. The retina was attached but the impaired visual acuity of the right eye was not improved [110].

Our literature search did not yield any reports of ophthalmic adverse events in response to other anti-CD20 mAbs, such as ofatumumab, ocrelizumab, and ublituximab.

#### Ocular ADR to bsDMARDs

Currently available bsDMARDs encompass adalimumab, etanercept, infliximab, and RTX (Table 1), all of which have received regulatory approval following the demonstration that their clinical properties did not significantly differ from those of their biological originators in terms of tolerance, efficacy, safety, pharmacodynamics, and immunogenicity [111, 112]. The same evidence-based recommendations can be applied when switching from bio-originators to biosimilars or vice versa, and among biosimilars,

The introduction of biosimilars has greatly expanded access to effective therapeutic agents for all RA patients with disease recalcitrant to csDMARDs and tsDMARDs, although the cost of these drugs remains high. To the best of our knowledge, no clinically meaningful ocular adverse events different from those linked to their originators have been reported in RA patients thus far.

## Discussion

Although a chronic, incurable, lifelong disease, RA can be controlled and substantially improved in the large majority, if not all patients, given the current therapeutic armamentarium. However, the long-term use of these drugs, even at the recommended doses, is inevitably associated with a risk of severe adverse events that can potentially involve the eye. Ocular toxicity induced by DMARDs in patients with RA is not particularly rare, especially given the more aggressive and combination regimens. As the use of these agents increases, the occurrence of adverse events, while remaining rare, can be expected to increase. In the case of ocular toxicity, the mechanism(s) are for the most part poorly understood. In addition, in the absence of biomarkers indicative of the potential risk of ophthalmological complications, ocular toxicities induced by DMARDs are poorly preventable. Consequently, an accurate risk vs. benefit appraisal of these therapies should be made for each patient. With increasingly sophisticated pharmacogenomics, the management of RA will likely one day include the identification of polymorphisms associated with variations in treatment response or toxicity [113].

In our literature search, most of the ocular ADRs were described in case reports and case series, summarized in Tables 4 and 5. Although a wide spectrum of ADRs have been described in RA patients, the actual prevalence is probably higher and the clinical manifestations more heterogeneous than can be inferred from the literature, as an undefined but non-negligible number of ADRs likely remain undiagnosed or unreported. Randomized, controlled studies are crucial in establishing, with reasonable likelihood, a causal relationship between drug administration and the onset of an untoward clinical event. According to the causality assessment scale [18], the level of evidence for most of the drug reactions described in the present review is low, restricted to the “probable/likely” or “possible” rather than to the “certain” category (Table 3). Moreover, because most ADRs are non-specific, it is often difficult to distinguish them from the clinical manifestations of the underlying disease. Dechallenge/rechallenge or dose/response tests, which can help identify ADRs, have been reported only in a minority of publications. Nonetheless, until ocular ADRs are confirmed by additional observations over time, clinicians should be mindful of their risk and promptly report those detected in their patients to the scientific and medical communities.

The cause/effect relationship between GC-induced PSC and glaucoma is undisputable. PSC development is related not only to the dose and length of GC administration but also to the patient’s susceptibility [114], thus challenging the notion of a safe dose and explaining the wide-ranging incidence of 6% to almost 40% [24]. Secondary glaucoma may develop regardless of whether GCs are administered orally, intravenously, or topically. Although glycosaminoglycan accrual in the trabecular meshwork seems to play a major role in PSC, an association between mutations in the gene encoding the trabecular meshwork protein and open-angle glaucoma has been determined in a minority of patients taking GCs [33, 115].

In the USA, approximately one million cases of HZ are annually diagnosed, 10% of which are subtyped as ophthalmic HZ [116]. Patients with RA are known to be at risk for developing HZ, including ophthalmic HZ and post-herpetic neuralgia [117]. Clinical experience has shown that this baseline risk is enhanced by drugs such as JAK inhibitors (mostly tofacitinib), TNFis, and IL-6Ris (mostly infliximab), especially when used in combination with GCs or in patients with co-morbidities or 50 years of age or older [118]. The mechanisms underlying the increased risk of VZV reactivation in these patients are unclear, but a possible explanation is that the drugs interfere with the onset and persistence of VZV-specific memory T-cells [58].

The obvious implication of these observations is that, before starting therapies capable of reactivating VZV, RA patients should be vaccinated with an anti-HZ vaccine. The live attenuated vaccine (Zostavax®, Merck Sharp and Dohme) has, however, found poor reception among rheumatologists because, while effectively preventing shingles, it may be problematic in patients immunocompromised by biologic agents [58]. A two-dose adjuvanted recombinant subunit, and hence non-live, anti-HZ vaccine (Shingrix®, GlaxoSmithKline) is equally if not more effective and may thus be more appropriate for RA patients considered to be at a higher risk of VZV reactivation [119]. This vaccine has also been recommended for the general population by the Advisory Committee on Immunization Practices for Use of Herpes Zoster Vaccines [120].

The development of sarcoidosis during TNFis treatment is a paradoxical, non-rare, and under-recognized adverse event. Although most cases involved etanercept, this ADR has been found to occur with all TNFis, suggesting a class- rather than a drug-specific effect [66, 121, 122]. Discontinuation of anti-TNF treatment, the administration of GCs, or both have, in the large majority of cases, resulted in at least partial resolution of the sarcoid lesions whereas reintroduction of the anti-TNF led to relapse [122]. Additional evidence that granulomatosis is directly related to TNF blocker therapy comes from a retrospective study of 2,800 patients who were treated with TNFis in 2008 in France. Sarcoidosis developed in at least 1 patient (0.04%), a prevalence higher than the country’s annual incidence of sarcoidosis (6 per 100,000: 0.006%) [69].

The mechanisms whereby TNFis induce sarcoid-like lesions are unknown. An inverse and interdependent relationship between TNF-α and interferon levels, and the increase in interferon levels that characterizes the onset of autoimmune diseases have been proposed. Additional explanations are the disruption of the fine balance of cytokines involved in granuloma formation, or the ability of TNFis to promote infections by microbes that induce noncaseating granuloma formation [123].

The ocular ADRs summarized in Table 5 support a link between TNF-α inhibition and the occurrence of demyelinating and central nervous system events. Typical examples are the development of ON [83–89] but also in rare instances of oculomotor palsy [93] and chiasmopathy [92] following the administration of TNFis in RA patients. Obviously, caution must be exercised before assuming causality, given the difficulty in establishing whether the demyelinating events are: a) truly of new onset and directly caused by the TNFi, b) the unmasking and enhancing of a latent multiple sclerosis that, in the absence of the drug, would have become manifest at a later time, or c) a simple coincidence of TNFi administration and the appearance of demyelinating disease [124]. It has been suggested that TNF-α antagonists directly alter the immune response and increase autoimmune activity, thus enhancing demyelination [125]. Despite the uncertainties regarding this association, if the clinical evaluation leads to the diagnosis of ON, the TNFi should be discontinued, replaced by steroid treatment. Overall, anti-TNF agents should be considered as contraindicated in patients with established demyelinating disease.

The ADRs reported in the literature and described herein reaffirm the notion that multimorbidity, including visual function impairment, is common in RA, and its frequency increases significantly in step with polypharmacy. Thus, holistic care and vigilance in the detection of medication-induced ocular adverse events are critical aspects in the clinical management of RA patients. Prompt consultation between an ophthalmologist well aware of these potential complications and a rheumatologist or an internist with appropriate expertise can lead to early detection, proper diagnosis, and treatment to prevent or substantially reduce the severity and duration of ocular ADRs.

## Data Availability

Data are available upon justified request.

## Abbreviations

ADRs: Adverse drug reactions
AU: Anterior uveitis
bDMARDs: Biological disease-modifying antirheumatic drugs
bsDMARDs: Biosimilar disease-modifying antirheumatic drugs
CDAI: Clinical disease activity index
csDMARDs: Conventional synthetic disease-modifying antirheumatic drugs
DMARDs: Disease-modifying antirheumatic drugs
EULAR: European League Against Rheumatism
GCs: Glucocorticoids
HCQ: Hydroxychloroquine
HL: Hodgkin’s lymphoma
HZ: Herpes zoster
IL-6R-is: Interleukin-6 receptor inhibitors
IOP: Intraocular pressure
JAK–STAT: Janus kinase–signal transducers and activators of transcription
KCS: Keratoconjunctivitis sicca
LDA: Low disease activity
mAb: Monoclonal antibody
MALT: Mucosa-associated lymphoid tissue
MTX: Methotrexate
NHL: Non-Hodgkin's lymphoma
NSAIDs: Non-steroidal anti-inflammatory drugs
ON: Optic neuritis
PSCs: Posterior subcapsular cataracts
PUK: Peripheral ulcerative keratitis
RA: Rheumatoid arthritis
RTX: Rituximab
TCZ: Tocilizumab
TNF: Tumor necrosis factor
TNFi: Tumor necrosis factor inhibitor
tsDMARDs: Targeted synthetic disease-modifying antirheumatic drugs
VK: Vortex keratopathy
VZV: Varicella zoster virus
WHO: World Health Organization

## References

[1] Aletaha D, Smolen JS (2018). Diagnosis and management of rheumatoid arthritis: a review. JAMA.

[2] Rossini M, Rossi E, Bernardi D (2014). Prevalence and incidence of rheumatoid arthritis in Italy. Rheumatol Int.

[3] Galloway J, Capron JP, De Leonardis F (2020). The impact of disease severity and duration on cost, early retirement and ability to work in rheumatoid arthritis in Europe: an economic modelling study. Rheumatol Adv Pract..

[4] Smolen JS, Landewé R, Breedveld FC (2010). EULAR recommendations for the management of rheumatoid arthritis with synthetic and biological disease-modifying antirheumatic drugs. Ann Rheum Dis.

[5] Smolen JS, Landewé RBM, Bijlsma JWJ (2020). EULAR recommendations for the management of rheumatoid arthritis with synthetic and biological disease-modifying antirheumatic drugs: 2019 update. Ann Rheum Dis.

[6] Felson DT, Smolen JS, Wells G (2011). American College of Rheumatology/European League against Rheumatism provisional definition of remission in rheumatoid arthritis for clinical trials. Ann Rheum Dis.

[7] Aletaha D, Nell VP, Stamm T (2005). Acute phase reactants add little to composite disease activity indices for rheumatoid arthritis: validation of a clinical activity score. Arthritis Res Ther.

[8] Larizgoitia I, Bouesseau MC, Kelley E (2013). WHO Efforts to Promote Reporting of Adverse Events and Global Learning. J Public Health Res..

[9] Freitas R, Godinho F, Madeira N (2020). Safety and effectiveness of biologic disease-modifying antirheumatic drugs in older patients with rheumatoid arthritis: a prospective cohort study. Drugs Aging.

[10] Turk MA, Hayworth JL, Nevskaya T, Pope JE (2021). Ocular manifestations in rheumatoid arthritis, connective tissue disease, and vasculitis: a systematic review and metaanalysis. J Rheumatol.

[11] Bhamra MS, Gondal I, Amarnani A (2019). Ocular manifestations of rheumatoid arthritis: implications of recent clinical trials. Int J Clin Res Trials.

[12] Kemeny-Beke A, Szodoray P (2020). Ocular manifestations of rheumatic diseases. Int Ophthalmol.

[13] Cimmino MA, Salvarani C, Macchioni P (2000). Extra-articular manifestations in 587 Italian patients with rheumatoid arthritis. Rheumatol Int.

[14] Conforti A, Di Cola I, Pavlych V (2021). Beyond the joints, the extra-articular manifestations in rheumatoid arthritis. Autoimmun Rev..

[15] Smolen JS, Aletaha D (2015). Rheumatoid arthritis therapy reappraisal: strategies, opportunities and challenges. Nat Rev Rheumatol.

[16] Naranjo CA, Busto U, Sellers EM (1981). A method for estimating the probability of adverse drug reactions. Clin Pharmacol Ther.

[17] Moorthy RS, Valluri S (1999). Ocular toxicity associated with systemic drug therapy. Curr Opin Ophthalmol.

[18] Fraunfelder FW, Fraunfelder FT (2004). Adverse ocular drug reactions recently identified by the national registry of drug-induced ocular side effects. Ophthalmology.

[19] Mortada A, Abboud I (1973). Retinal haemorrhages after prolonged use of salicylates. Br J Ophthalmol.

[20] Black RA, Bensinger RE (1982). Bilateral subconjunctival hemorrhage after acetylsalicylic acid overdose. Ann Ophthalmol.

[21] Groomer AE, Terry JE, Westblom TU (1990). Subconjunctival and external hemorrhage secondary to oral anticoagulation. J Am Optom Assoc.

[22] Burns CA (1968). Indomethacin, reduced retinal sensitivity, and corneal deposits. Am J Ophthalmol.

[23] Palimeris G, Koliopoulos J, Velissaropoulos P (1972). Ocular side effects of indomethacin. Ophthalmologica.

[24] Peponis V, Kyttaris VC, Chalkiadakis SE, Bonovas S, Sitaras NM (2010). Ocular side effects of anti-rheumatic medications: what a rheumatologist should know. Lupus.

[25] Henkes HE, van Lith GH, Canta LR (1972). Indomethacin retinopathy. Am J Ophthalmol.

[26] Graham CM, Blach RK (1988). Indomethacin retinopathy: case report and review. Br J Ophthalmol.

[27] Landewé RB, Boers M, Verhoeven AC (2002). COBRA combination therapy in patients with early rheumatoid arthritis: long-term structural benefits of a brief intervention. Arthritis Rheum.

[28] Chatzidionysiou K, Emamikia S, Nam J (2017). Efficacy of glucocorticoids, conventional and targeted synthetic disease-modifying antirheumatic drugs: a systematic literature review informing the 2016 update of the EULAR recommendations for the management of rheumatoid arthritis. Ann Rheum Dis.

[29] Crossfield SSR, Buch MH, Baxter P, Kingsbury SR, Pujades-Rodriguez M, Conaghan PG (2021). Changes in the pharmacological management of rheumatoid arthritis over two decades. Rheumatology (Oxford).

[30] Wilson JC, Sarsour K, Gale S, Pethö-Schramm A, Jick SS, Meier CR (2019). Incidence and risk of glucocorticoid-associated adverse effects in patients with rheumatoid arthritis. Arthritis Care Res (Hoboken).

[31] Black RL, Oglesby RB, Von Sallmann L, Bunim JJ (1960). Posterior subcapsular cataracts induced by corticosteroids in patients with rheumatoid arthritis. JAMA.

[32] Urban RC, Cotlier E (1986). Corticosteroid-induced cataracts. Surv Ophthalmol.

[33] Stone EM, Fingert JH, Alward WL (1997). Identification of a gene that causes primary open angle glaucoma. Science.

[34] Liuska PJ, Harju M, Kivelä TT, Turunen JA (2021) Prevalence of MYOC risk variants for glaucoma in different populations. Acta Ophthalmol 2021 Jan 9 10.1111/aos.14738 33421356

[35] Rangachari K, Bankoti N, Shyamala N (2019). Glaucoma pred: glaucoma prediction based on Myocilin genotype and phenotype information. Genomics.

[36] Li J, Tripathi RC, Tripathi BJ (2008). Drug-induced ocular disorders. Drug Saf.

[37] Balachandran C, McCluskey PJ, Champion GD, Halmagyi GM (2002). Methotrexate-induced optic neuropathy. Clin Exp Ophthalmol.

[38] Clare G, Colley S, Kennett R, Elston JS (2005). Reversible optic neuropathy associated with low-dose methotrexate therapy. J Neuroophthalmol.

[39] van Ede AE, Laan RF, Blom HJ (2002). Homocysteine and folate status in methotrexate-treated patients with rheumatoid arthritis. Rheumatology (Oxford).

[40] Al-Tweigeri T, Nabholtz JM, Mackey JR (1996). Ocular toxicity and cancer chemotherapy: a review. Cancer.

[41] Klemencic S (2010). Cotton wool spots as an indicator of methotrexate-induced blood dyscrasia. Optometry.

[42] Mercer LK, Regierer AC, Mariette X (2017). Spectrum of lymphomas across different drug treatment groups in rheumatoid arthritis: a European registries collaborative project. Ann Rheum Dis.

[43] Klein A, Polliack A, Gafter-Gvili A (2018). Rheumatoid arthritis and lymphoma: Incidence, pathogenesis, biology, and outcome. Hematol Oncol.

[44] Kobayashi Y, Kimura K, Fujitsu Y, Shinkawa K, Muta H, Sonoda KH (2016). Methotrexate-associated orbital lymphoproliferative disorder in a patient with rheumatoid arthritis: a case report. Jpn J Ophthalmol.

[45] Barak A, Morse LS, Schwab I (2004). Leflunomide (Arava)-induced cystoid macular oedema. Rheumatology (Oxford).

[46] Hassikou H, El Haouri M, Tabache F, Baaj M, Safi S, Hadri L (2008). Leflunomide-induced toxic epidermal necrolysis in a patient with rheumatoid arthritis. Joint Bone Spine.

[47] Santodomingo-Rubido J, Gilmartin B, Wolffsohn JS (2003). Drug-induced bilateral transient myopia with the sulphonamide sulphasalazine. Ophthalmic Physiol Opt.

[48] Dammacco R (2018). Systemic lupus erythematosus and ocular involvement: an overview. Clin Exp Med.

[49] Dammacco R, Procaccio P, Racanelli V, Vacca A, Dammacco F (2018). Ocular involvement in systemic lupus erythematosus: the experience of two tertiary referral centers. Ocul Immunol Inflamm.

[50] Yusuf IH, Sharma S, Luqmani R, Downes SM (2017). Hydroxychloroquine retinopathy. Eye (Lond).

[51] Mavrikakis I, Sfikakis PP, Mavrikakis E (2003). The incidence of irreversible retinal toxicity in patients treated with hydroxychloroquine: a reappraisal. Ophthalmology.

[52] Marmor MF, Kellner U, Lai TY, Melles RB, Mieler WF (2016). American Academy of Ophthalmology: recommendations on screening for chloroquine and hydroxychloroquine retinopathy (2016 revision). Ophthalmology.

[53] Ikegawa Y, Shiraishi A, Hayashi Y, Ogimoto A, Ohashi Y (2018). In vivo confocal microscopic observations of vortex keratopathy in patients with amiodarone-induced keratopathy and fabry disease. J Ophthalmol.

[54] Au SCL (2020). Hydroxychloroquine retinal toxicity: the bull's eye in the human eye. Vis J Emerg Med.

[55] Banerjee S, Biehl A, Gadina M, Hasni S, Schwartz DM (2017). JAK-STAT signaling as a target for inflammatory and autoimmune diseases: current and future prospects. Drugs.

[56] Gadina M, Le MT, Schwartz DM (2019). Janus kinases to jakinibs: from basic insights to clinical practice. Rheumatology (Oxford).

[57] Mease P, Charles-Schoeman C, Cohen S (2020). Incidence of venous and arterial thromboembolic events reported in the tofacitinib rheumatoid arthritis, psoriasis and psoriatic arthritis development programmes and from real-world data. Ann Rheum Dis.

[58] Winthrop KL, Yamanaka H, Valdez H (2014). Herpes zoster and tofacitinib therapy in patients with rheumatoid arthritis. Arthritis Rheumatol.

[59] Yamaoka K (2016). Benefit and risk of tofacitinib in the treatment of rheumatoid arthritis: a focus on herpes zoster. Drug Saf.

[60] Smitten AL, Simon TA, Hochberg MC, Suissa S (2008). A meta-analysis of the incidence of malignancy in adult patients with rheumatoid arthritis. Arthritis Res Ther.

[61] Maini RN, Elliott M, Brennan FM, Williams RO, Feldmann M (1997). TNF blockade in rheumatoid arthritis: implications for therapy and pathogenesis. APMIS.

[62] Mewar D, Wilson AG (2011). Treatment of rheumatoid arthritis with tumour necrosis factor inhibitors. Br J Pharmacol.

[63] Chen AY, Wolchok JD, Bass AR (2021). TNF in the era of immune checkpoint inhibitors: friend or foe?. Nat Rev Rheumatol.

[64] Gaggiano C, Sota J, Gentileschi S (2020). The current status of biological treatment for uveitis. Expert Rev Clin Immunol.

[65] Lim LL, Fraunfelder FW, Rosenbaum JT (2007). Do tumor necrosis factor inhibitors cause uveitis? A registry-based study. Arthritis Rheum.

[66] Koda K, Toyoshima M, Nozue T, Suda T (2020). Systemic sarcoidosis associated with certolizumab pegol treatment for rheumatoid arthritis: a case report and review of the literature. Intern Med.

[67] Sturfelt G, Christensson B, Bynke G, Saxne T (2007). Neurosarcoidosis in a patient with rheumatoid arthritis during treatment with infliximab. J Rheumatol.

[68] Suzuki J, Goto H (2009). Uveitis associated with sarcoidosis exacerbated by etanercept therapy. Jpn J Ophthalmol.

[69] Daïen CI, Monnier A, Claudepierre P (2009). Club Rhumatismes et inflammation (CRI): sarcoid-like granulomatosis in patients treated with tumor necrosis factor blockers: 10 cases. Rheumatology (Oxford).

[70] Clementine RR, Lyman J, Zakem J, Mallepalli J, Lindsey S, Quinet R (2010). Tumor necrosis factor-alpha antagonist-induced sarcoidosis. J Clin Rheumatol.

[71] Seve P, Varron L, Broussolle C, Denis P, Kodjikian L (2012). Sarcoid-related uveitis occurring during adalimumab therapy. Ocul Immunol Inflamm.

[72] Dragnev D, Barr D, Kulshrestha M, Shanmugalingam S (2013). Sarcoid panuveitis associated with etanercept treatment, resolving with adalimumab. BMJ Case Rep.

[73] Durel CA, Feurer E, Pialat JB, Berthoux E, Chapurlat RD, Confavreux CB (2013). Etanercept may induce neurosarcoidosis in a patient treated for rheumatoid arthritis. BMC Neurol.

[74] Moisseiev E, Shulman S (2014). Certolizumab-induced uveitis: a case report and review of the literature. Case Rep Ophthalmol.

[75] Berrios I, Jun-O’Connell A, Ghiran S, Ionete C (2015). A case of neurosarcoidosis secondary to treatment of etanercept and review of the literature. BMJ Case Rep.

[76] Isshiki T, Matsuyama H, Sakamoto S (2019). Development of propionibacterium acnes-associated sarcoidosis during etanercept therapy. Intern Med.

[77] Sobolewska B, Baglivo E, Edwards AO (2021). Drug-induced sarcoid uveitis with biologics. Ocul Immunol Inflamm.

[78] Wendling D, Paccou J, Berthelot JM (2011). CRI: new onset of uveitis during anti-tumor necrosis factor treatment for rheumatic diseases. Semin Arthritis Rheum..

[79] Tiliakos AN, Tiliakos NA (2003). Ocular inflammatory disease in patients with RA taking etanercept: is discontinuation of etanercept necessary?. J Rheumatol.

[80] Fonollosa A, Segura A, Giralt J, Garcia-Arumi J (2007). Tuberculous uveitis after treatment with etanercept. Graefes Arch Clin Exp Ophthalmol.

[81] Burns AM, Green PJ, Pasternak S (2012). Etanercept-induced cutaneous and pulmonary sarcoid-like granulomas resolving with adalimumab. J Cutan Pathol.

[82] Prasse A, Georges CG, Biller H (2000). Th1 cytokine pattern in sarcoidosis is expressed by bronchoalveolar CD4+ and CD8+ T cells. Clin Exp Immunol.

[83] Dammacco R, Biswas J, Kivelä TT (2020). Ocular sarcoidosis: clinical experience and recent pathogenetic and therapeutic advancements. Int Ophthalmol.

[84] Jung JH, Kim JH, Song GG (2017). Adalimumab-induced pulmonary sarcoidosis not progressing upon treatment with etanercept. Z Rheumatol.

[85] Foroozan R, Buono LM, Sergott RC, Savino PJ (2002). Retrobulbar optic neuritis associated with infliximab. Arch Ophthalmol.

[86] ten Tusscher MP, Jacobs PJ, Busch MJ, de Graaf L, Diemont WL (2003). Bilateral anterior toxic optic neuropathy and the use of infliximab. BMJ.

[87] Tran TH, Milea D, Cassoux N, Bodaghi B, Bourgeois P, LeHoang P (2005). Névrite optique rétrobulbaire associée au traitement par infliximab [Optic neuritis associated with infliximab]. J Fr Ophtalmol.

[88] Simsek I, Erdem H, Pay S, Sobaci G, Dinc A (2007). Optic neuritis occurring with anti-tumour necrosis factor alpha therapy. Ann Rheum Dis.

[89] Noguera-Pons R, Borrás-Blasco J, Romero-Crespo I, Antón-Torres R, Navarro-Ruiz A, González-Ferrandez JA (2005). Optic neuritis with concurrent etanercept and isoniazid therapy. Ann Pharmacother.

[90] Chung JH, Van Stavern GP, Frohman LP, Turbin RE (2006). Adalimumab-associated optic neuritis. J Neurol Sci.

[91] Sicotte NL, Voskuhl RR (2001). Onset of multiple sclerosis associated with anti-TNF therapy. Neurology.

[92] Farrell E, Sacks JG (2008). Infliximab-associated Chiasmopathy. Ochsner J.

[93] Farukhi FI, Bollinger K, Ruggieri P, Lee MS (2006). Infliximab-associated third nerve palsy. Arch Ophthalmol.

[94] Roos JC, René C, Ostor AJ (2011). Necrotizing group A streptococcal periorbital infection following adalimumab therapy for rheumatoid arthritis. Cutan Ocul Toxicol.

[95] Caramaschi P, Biasi D, Carletto A, Bambara LM (2003). Orbital myositis in a rheumatoid arthritis patient during etanercept treatment. Clin Exp Rheumatol.

[96] Couderc M, Mathieu S, Tournadre A, Dubost JJ, Soubrier M (2014). Acute ocular myositis occurring under etanercept for rheumatoid arthritis. Joint Bone Spine.

[97] Damento G, Kavoussi SC, Materin MA (2014). Clinical and histologic findings in patients with uveal melanomas after taking tumor necrosis factor-α inhibitors. Mayo Clin Proc.

[98] Diniz B, Barbosa CP, Regatieri CV, Rodrigues EB (2011). Oclusão de ramo venoso da retina associado ao uso de infliximabe: relato de caso [Branch retinal vein occlusion following infliximab treatment: case report]. Arq Bras Oftalmol.

[99] Raaschou P, Simard JF, Holmqvist M, Askling J (2013). ARTIS Study Group: rheumatoid arthritis, anti-tumour necrosis factor therapy, and risk of malignant melanoma: nationwide population based prospective cohort study from Sweden. BMJ.

[100] Smith KJ, Skelton HG (2001). Rapid onset of cutaneous squamous cell carcinoma in patients with rheumatoid arthritis after starting tumor necrosis factor alpha receptor IgG1-Fc fusion complex therapy. J Am Acad Dermatol.

[101] Elinav E, Nowarski R, Thaiss CA, Hu B, Jin C, Flavell RA (2013). Inflammation-induced cancer: crosstalk between tumours, immune cells and microorganisms. Nat Rev Cancer.

[102] Nouri B, Nair N, Barton A (2020). Predicting treatment response to IL6R blockers in rheumatoid arthritis. Rheumatology (Oxford).

[103] Tada A, Hashida N, Tanaka T, Nishida K (2012). Anti-interleukin-6 receptor antibody therapy-induced retinopathy in a patient with rheumatoid arthritis. Case Rep Rheumatol..

[104] Roux C, Breuil V, Albert C (2011). Ophthalmic herpes zoster infection in patients with rheumatoid arthritis who were treated with tocilizumab. J Rheumatol.

[105] Heissigerová J, Callanan D, de Smet MD (2019). Efficacy and safety of sarilumab for the treatment of posterior segment noninfectious uveitis (SARIL-NIU): the phase 2 SATURN study. Ophthalmology.

[106] Smolen JS, Tohidast-Akrad M, Gal A (1996). The role of T-lymphocytes and cytokines in rheumatoid arthritis. Scand J Rheumatol.

[107] Ruderman EM, Pope RM (2006). Drug insight: abatacept for the treatment of rheumatoid arthritis. Nat Clin Pract Rheumatol.

[108] Tsuboi H, Matsumoto I, Hagiwara S (2015). Efficacy and safety of abatacept for patients with Sjögren's syndrome associated with rheumatoid arthritis: rheumatoid arthritis with orencia trial toward Sjögren's syndrome endocrinopathy (ROSE) trial-an open-label, one-year, prospective study-Interim analysis of 32 patients for 24 weeks. Mod Rheumatol.

[109] Puéchal X, Gottenberg JE, Berthelot JM (2012). Investigators of the autoImmunity rituximab registry. Rituximab therapy for systemic vasculitis associated with rheumatoid arthritis: results from the AutoImmunity and rituximab registry. Arthritis Care Res (Hoboken).

[110] Schuler S, Brunner M, Bernauer W (2016). Rituximab and acute retinal necrosis in a patient with scleromalacia and rheumatoid arthritis. Ocul Immunol Inflamm.

[111] Kay J, Schoels MM, Dörner T (2018). Task force on the use of biosimilars to treat rheumatological diseases. Consensus-based recommendations for the use of biosimilars to treat rheumatological diseases. Ann Rheum Dis.

[112] Melville AR, Md Yusof MY, Fitton J (2021). Real-world experience of effectiveness of non-medical switch from originator to biosimilar rituximab in rheumatoid arthritis. Rheumatology (Oxford)..

[113] Dedmon LE (2020). The genetics of rheumatoid arthritis. Rheumatology (Oxford).

[114] Skalka HW, Prchal JT (1980). Effect of corticosteroids on cataract formation. Arch Ophthalmol.

[115] Yemanyi F, Vranka J, Raghunathan VK (2020). Glucocorticoid-induced cell-derived matrix modulates transforming growth factor β2 signaling in human trabecular meshwork cells. Sci Rep.

[116] Davis AR, Sheppard J (2019). Herpes zoster ophthalmicus review and prevention. Eye Contact Lens.

[117] Kawai K, Yawn BP (2017). Risk factors for herpes zoster: a systematic review and meta-analysis. Mayo Clin Proc.

[118] Riley TR, George MD (2021). Risk for infections with glucocorticoids and DMARDs in patients with rheumatoid arthritis. RMD Open.

[119] Lecrenier N, Beukelaers P, Colindres R (2018). Development of adjuvanted recombinant zoster vaccine and its implications for shingles prevention. Expert Rev Vaccines.

[120] Dooling KL, Guo A, Patel M (2018). Recommendations of the advisory committee on immunization practices for use of herpes zoster vaccines. MMWR Morb Mortal Wkly Rep.

[121] Massara A, Cavazzini L, La Corte R, Trotta F (2010). Sarcoidosis appearing during anti-tumor necrosis factor alpha therapy: a new “class effect” paradoxical phenomenon. Two case reports and literature review. Semin Arthritis Rheum.

[122] Tong D, Manolios N, Howe G, Spencer D (2012). New onset sarcoid-like granulomatosis developing during anti-TNF therapy: an under-recognised complication. Intern Med J.

[123] Nicolela Susanna F, Pavesio C (2020). A review of ocular adverse events of biological anti-TNF drugs. J Ophthalmic Inflamm Infect.

[124] Magnano MD, Robinson WH, Genovese MC (2004). Demyelination and inhibition of tumor necrosis factor (TNF). Clin Exp Rheumatol.

[125] Robinson WH, Genovese MC, Moreland LW (2001). Demyelinating and neurologic events reported in association with tumor necrosis factor alpha antagonism: by what mechanisms could tumor necrosis factor alpha antagonists improve rheumatoid arthritis but exacerbate multiple sclerosis?. Arthritis Rheum.

